# Molecular Structure of Nickel Octamethylporphyrin—Rare Experimental Evidence of a Ruffling Effect in Gas Phase

**DOI:** 10.3390/ijms23010320

**Published:** 2021-12-28

**Authors:** Alexander E. Pogonin, Arseniy A. Otlyotov, Yury Minenkov, Alexander S. Semeikin, Yuriy A. Zhabanov, Sergey A. Shlykov, Georgiy V. Girichev

**Affiliations:** 1 Department of Nanomaterials and Ceramic Technology, Ivanovo State University of Chemistry and Technology, Sheremetevsky Avenue 7, 153000 Ivanovo, Russia; 2 Department of Physics, Ivanovo State University of Chemistry and Technology, Sheremetevsky Avenue 7, 153000 Ivanovo, Russia; arseney_otlyotov@mail.ru (A.A.O.); zhabanov@gmail.com (Y.A.Z.); 3 N.N. Semenov Institute of Chemical Physics of Russian Academy of Sciences, Kosygina Street 4, 119991 Moscow, Russia; Yury.Minenkov@chph.ras.ru; 4 Joint Institute for High Temperatures, Russian Academy of Sciences, 13-2 Izhorskaya Street, 125412 Moscow, Russia; 5 Department of Organic Chemistry, Ivanovo State University of Chemistry and Technology, Sheremetevsky Avenue 7, 153000 Ivanovo, Russia; semeikin@isuct.ru; 6 Department of Physical and Colloidal Chemistry, Ivanovo State University of Chemistry and Technology, Sheremetevsky Avenue 7, 153000 Ivanovo, Russia; shlykov@isuct.ru

**Keywords:** molecular structure, ruffling distortion, porphyrin, quantum chemistry, nickel, electron diffraction

## Abstract

The structure of a free nickel (II) octamethylporphyrin (NiOMP) molecule was determined for the first time through a combined gas-phase electron diffraction (GED) and mass spectrometry (MS) experiment, as well as through quantum chemical (QC) calculations. Density functional theory (DFT) calculations do not provide an unambiguous answer about the planarity or non-planar distortion of the NiOMP skeleton. The GED refinement in such cases is non-trivial. Several approaches to the inverse problem solution were used. The obtained results allow us to argue that the ruffling effect is manifested in the NiOMP molecule. The minimal critical distance between the central atom of the metal and nitrogen atoms of the coordination cavity that provokes ruffling distortion in metal porphyrins is about 1.96 Å.

## 1. Introduction

Porphyrin metal complexes are the active core of many biochemical systems, e.g., hemoglobin, chlorophyll and vitamin B_12_ [1,2]. An important source of porphyrins is oil, a ton of which can contain up to several kilograms of porphyrins, especially vanadyl-ion (VO^2+^) and nickel ion (Ni^2+^) complexes [2].

Porphyrin metal complexes are the active catalysts in chemical, electrochemical and photochemical processes, including the electroreduction of molecular oxygen, the anodic oxidation of sulfur dioxide, the cathodic reduction of nitrous oxide, the isomerization of unsaturated compounds, etc. [3]. Some etioporphyrin metal complexes are now considered to be potential radioprotective agents and stimulants of post-radiation rehabilitation [3]. Moreover, porphyrin metal complexes are promising agents for photodynamic therapy and in the production of basic film materials for photovoltaic devices [4,5], microelectronics [6,7] and electrochemical Gratzel cells (DSSC) [8]. A number of physico-chemical properties of porphyrin compounds that determine the features of their absorption and luminescent spectra should be taken into account in the screening of effective sensibilizators [9]. Theoretical calculations of electronic transitions and the HOMO-LUMO energy gap are currently being performed in order to discover substances that are potentially effective in solar batteries [10,11]. Due to their stability at high temperatures, porphyrin metal complexes can be used as dyes under extreme conditions [2].

Porphyrin metal complexes are important for environmental protection as they can serve as sensible chemical materials in the new generation of resistive gas sensors because they possess improved metrological characteristics [12]. The influence of a central metal atom on the gas-sensitive properties of porphyrin-based materials was studied by examining the etioporphyrin (EP) complexes of cobalt, nickel, copper, zinc, palladium and platinum [12,13,14,15]. A sensor performance depends not only on the individual characteristics of the molecules, but on the structure of sprayed layers obtained through thermal sublimation as well [12].

The structural investigations of metal porphyrins are dominated by density functional theory (DFT) calculations. As many transition metal species are of a multireference character, the applicability of a single determinant DFT approach is questioned for porphyrin metal complexes. Nevertheless, our recent study [16] concludes that the ground state of nickel(II) complex with tetrakis(1,2,5-thiadiazolo)porphyrazine is clearly single-reference. This suggests that the DFT approach is most likely valid for other nickel(II) macroheterocycles.

**Figure 1:**
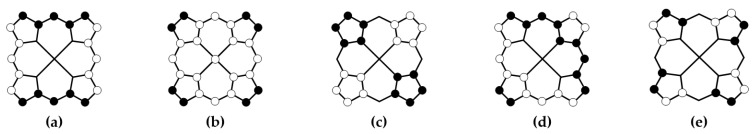
Possible types of porphyrin skeleton distortions: (**a**)—ruffling, (**b**)—dome shaped, (**c**)—saddle shaped, (**d**)—wave shaped, (**e**)—propeller shaped. A top view perpendicular to the macrocycle plane shows the positions of atoms relative to the plane of the porphyrin core: the light circle denotes an atom located above the plane, the dark circle denotes an atom located below the plane and the absence of a circle denotes the location of the atom in the plane of the porphyrin core.

The solid-state structures of porphyrin metal complexes are commonly obtained from X-ray diffraction experiments. According to a thorough study of the structure of porphyrin metal complexes [17,18], the distance from the center of the macrocycle cavity to the nitrogen atom of 2.01 Å corresponds to the minimal steric strain of the porphyrin core. Strong deviations from these values occurring in the case of complexes that possess a metal ionic radius that does not correspond to the cavity size results in different types of macroheterocycle distortions. Another factor is responsible for the deformations that are originated in various substituents located on the periphery of the molecule [19,20]. Under distortions, the spatial crowding of bulky substituents decreases, leading to the minimization of the peripheral steric strain. Figure 1 demonstrates the main typical distortions of the porphyrin cycle generally described in terms of normal coordinates. The study of ruffling, saddling and doming distortions is especially important in connection with their manifestation in biological molecules [21]. An explicit correlation between macrocycle nonplanarity and physico-chemical properties has been noted in ref. [22].

Some factors affecting the manifestation of the ruffling distortion were analyzed [23] for porphyrin complexes with relatively small central ions, such as Si(IV), P(V), Ge(IV) and As (V). The threshold between the flat and distorted structures was concluded to correspond to the distance between the central atom and the nitrogen atoms of 2.00–2.02 Å. The main structural parameter characterizing the degree of ruffling distortion is the dihedral angle χ(C_α_-N∙∙∙N-C_α_). In this case, a distortion of 20–30° is often accompanied by small energy changes (less than 4 kJ∙mol^−1^) indicating the extreme softness of this type of deformation. The authors of [23] also noted other structural parameters that change with the manifestation of the ruffling distortion of the macrocycle. For example, with increasing macrocyclic distortion, the bond angle C_α_-C_m_-C_α_ tends to decrease, while the C_β_-C_β_ distance and the C_α_-N-C_α_ angle tend to increase.

The DFT study of changes in the internal cavity within the series of the simplest porphyrin complexes MP (M = Cr, Mn, Fe, Co, Ni, Cu, Zn) was carried out in ref. [24]. Short M-N distances (typical for nickel complexes) lead to ruffling distortion. The authors of [24] also consider the dependence of the size of the coordination cavity on the number of electrons of the metal atom, which can be explained in terms of the population of d-orbitals. The largest cavity size is observed in the case of the MnP that possesses a high spin d^5^ state. The addition or removal of an electron (Fe an Cr complexes) results in a decrease in the cavity size due to the absence of an electron on the d_x2-y2_ orbital. The shortest M-N bonds were found for the nickel porphyrin (NiP) singlet state, where eight electrons occupy four d-orbitals, except for d_x2-y2_. In this case, the addition or removal of one electron (Cu, Co) leads to the enlargement of the MN_4_ fragment. The diamagnetic nature of nickel and zinc porphyrins are also experimentally well established [25,26,27].

According to the literature, nickel porphyrins exist in either flat [28,29,30,31] or ruffling-distorted [32,33,34] forms. In the triclinic crystal lattice, a molecule of nickel octaethylporphyrin (NiOEP) possesses a planar structure with Ni-N distances in the range of 1.946–1.958 Å [28,29], while in the tetragonal lattice a distorted structure with r(Ni-N) = 1.929 Å was found [34]. According to X-ray diffraction (XRD) and Raman spectroscopy investigation [31], the NiP molecule possesses a planar structure both in the crystal and in the solution, but the flat and distorted structures coexist in solutions of NiOEP and *meso*-tetraphenylporphyrin (NiTPP) [31,35]. The work [31] concludes that the replacement of peripheral hydrogen atoms by another substituent shifts the conformational equilibrium towards a non-planar structure. The macrocycle distortions are manifested in a bathochromic shift in the Q band of the absorption spectra [36], but this effect is insignificantly small. In ref. [37] it was shown that with an increase in the level of macrocycle distortion, a decrease in the energy gap of LUMO-HOMO occurs. Several NiTPP derivatives exhibit the effect of the degree of distortion on the redox potentials of the compounds.

According to the quantum chemical (QC) study [38], the conformers of NiOEP, with different arrangements of ethyl groups, exhibit some degree of ruffling distortion. The Ni-N distance is in the range of 1.96–1.97 Å. The flat structure maximizes the π-overlap in the system and corresponds to large-sized coordination cavity. At the same time, the size of Ni^2+^ is too small to form strong Ni-N bonds without the distortion of the planar macrocycle structure. Therefore, the equilibrium structures of porphyrin complexes with nickel are forced to achieve a balance between energy gains due to the shortening of the Ni-N bond and losses due to ruffling distortion because of a decrease in the degree of delocalization. According to the calculations carried out in [38], the ruffling distortion of a macroheterocycle reduces the total energy by ca. 0.8 kJ∙mol^−1^.

In the literature, there is a clear lack of data on the structure of free molecules of porphyrin metal complexes, while a number of studies focused on the structural determinations of phthalocyanine complexes using the gas electron diffraction (GED) method [39,40,41,42]. As for the porphyrins, the data on the gas-phase structures of the following compounds are available: copper (II) octamethylporphyrin (CuOMP) [43], copper(II) etioporphyrin-II (CuEP-II) [44], tin(II) octamethylporphyrin (SnOMP) [45], zinc(II) etioporphyrin-II (ZnEP-II) [46] and cobalt(II) etioporphyrin-II (CoEP-II) [47]. Based on the experimental and theoretical results, it was concluded that the above-mentioned porphyrin complexes with zinc, copper and cobalt have a flat structure, despite the fact that r(M-N) in the series decreases by almost 0.07 Å from 2.042 (5) Å in ZnEP-II [46] to 1.976 (5) Å in CoEP-II [47], which is below the threshold value of 2.01 Å [18]. Therefore, experimental studies of the gas-phase structures of nickel porphyrins with even shorter M-N distances are important for checking for a ruffling effect manifestation in porphyrins in a gas phase [47].

**Figure 2:**
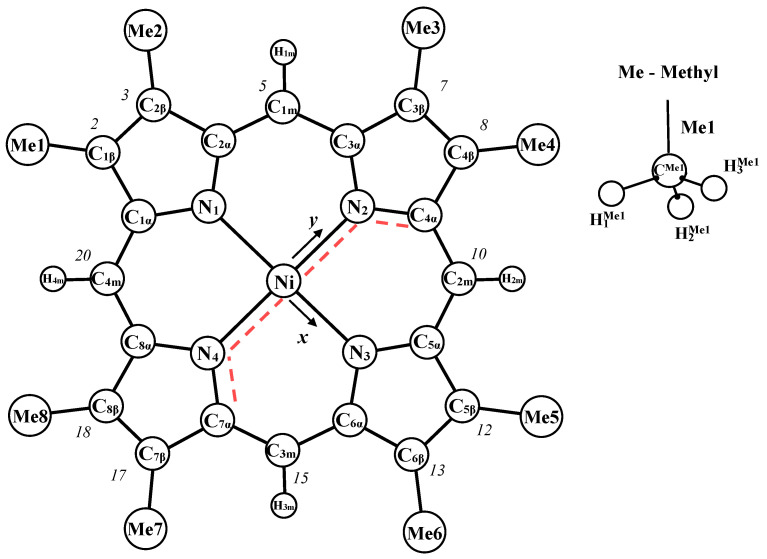
Molecular structure of NiOMP with atom numbering. The red dashed line shows an angle χ_ruf_ = χ(C_α_-N∙∙∙N-C_α_) quantifying the degree of ruffling distortion.

In the present contribution we report the results of the experimental GED and theoretical QC studies on nickel(II) 2,3,7,8,12,13,17,18-octamethylporphyrin (NiOMP, Figure 2).

## 2. Results

**Figure 3:**
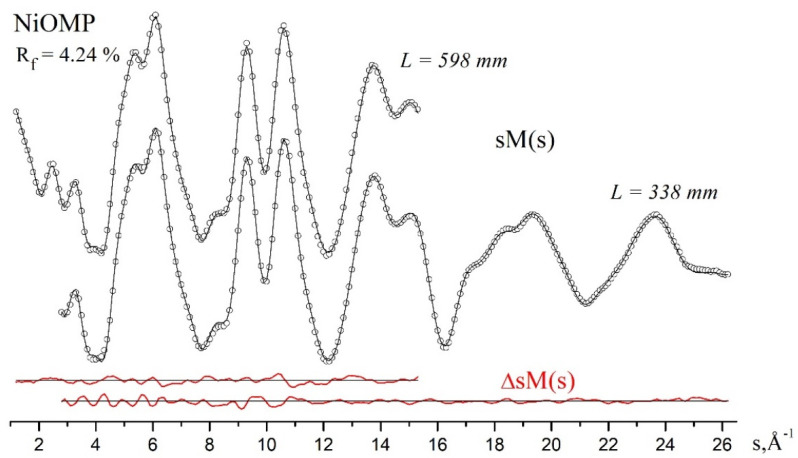
Experimental (cycles) and theoretical (black solid lines) molecular scattering intensities sM(s) and the difference curves ΔsM(s) (red solid lines) for NiOMP: disagreement factor R_f_ = 4.24%.

**Figure 4:**
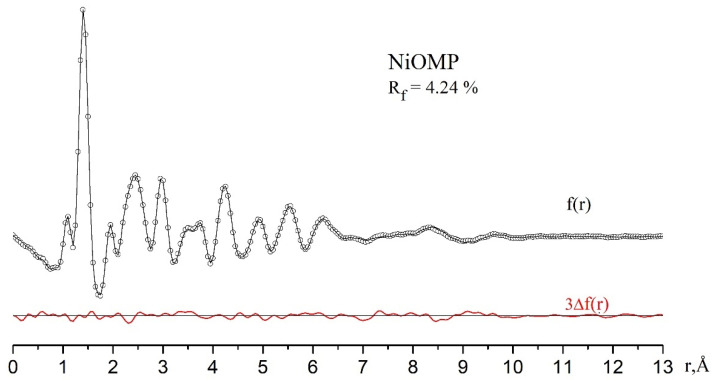
Experimental (cycles) and theoretical (black solid lines) radial distribution curves and the difference curve 3×Δf(r) (red solid lines) for NiOMP: disagreement factor R_f_ = 4.24%.

The optimized structures from the QC calculations are given in the Supplementary Materials. Experimental and theoretical molecular scattering intensities sM(s) for the two nozzle-to-film distances are shown in Figure 3, along with the corresponding difference curves. The radial distribution curves and the difference curve are shown in Figure 4. We highlight the non-trivial structural analysis detailed in Section 3.3 and Section 4.4.

## 3. Discussion

### 3.1. Ambiguity in NiOMP Molecular Structure

The performed QC calculations do not provide an unambiguous answer on the equilibrium spatial structure of the NiOMP molecule. The choice of the calculation level affects the prediction of the presence/absence of ruffling distortion in the macrocycle. Thus, according to the B3LYP calculations with the 6–31G * basis set, the NiOMP molecule possesses a non-planar geometric structure with a D_2d_ symmetry and a dihedral angle χ(C_α_-N N-C_α_) ≈ 24°, while the planar structure of the D_4h_ symmetry corresponds to the first-order saddle point on the potential energy surface (PES) and is 1.34 kJ∙mol^−1^ higher in energy compared to the non-planar model. The distance between the nickel and nitrogen atoms in the D_4h_ structure is larger than in the D_2d_ structure by 0.016 Å. The structural parameters of the macroheterocyclic skeleton only slightly change with the extension of the basis set, while the Ni-N distance increases by 0.02–0.03 Å, and the flat D_4h_ structure already corresponds to the minimum. The main structural parameters derived from the B3LYP calculations using various basis sets are listed in Table 1. It is important to note that the use of the relativistic core potential for the description of the inner electron shells (1s^2^2s^2^2p^6^) of the nickel atom does not lead to significant structural changes (Table 1).

**Figure 5:**
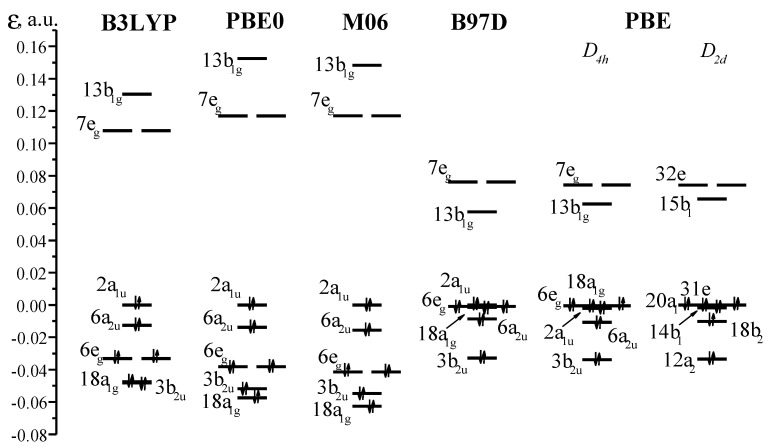
Molecular orbital diagrams for NiOMP according to different DFT methods. The energy of HOMO is chosen as zero level.

Since the calculations with the B3LYP functional did not provide an unambiguous answer on the presence/absence of a ruffling distortion in the NiOMP molecule, we expanded the range of DFT-functionals used (B97D, PBE0 and M06 in combination with the *cc-pVTZ* basis sets). According to the results of the calculations, the NiOMP molecule does not undergo a ruffling distortion. Considering the data given in Table 2, we note that in the series M06 → PBE0 → B3LYP → B97D → PBE, an increase in the related internuclear distances is observed, except for the Ni-N distance, which is smaller in the case of the PBE functional as compared to the B3LYP and B97D ones. It turned out that the set of theoretical methods listed above provides neither unique geometric nor electronic structures, predicting a different arrangement of frontier MOs (Figure 5).

**Figure 6:**
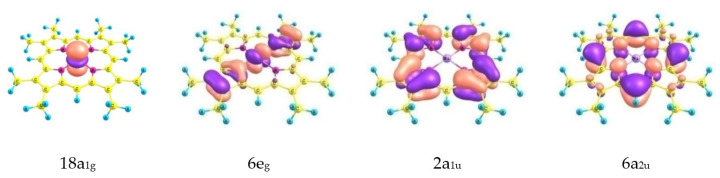
Molecular orbitals of NiOMP.

According to the results of the B3LYP, PBE0 and M06 calculations, HOMO and HOMO-1 are the MOs of the macrocycle of a_1u_ and a_2u_ symmetry (Figure 5). The doubly degenerate orbitals of e_g_ symmetry with a significant contribution (~30%) from the orbitals of the nickel atom (d_xz_, _yz_) are lower in energy by ~0.03 au. At the same time, according to PBE and B97D calculations, the lowest electronic state is characterized by the random degeneracy of the orbital of the 2a_1u_, MO 6e_g_ macrocycle with a contribution from the nickel atom of ~60% and 18a_1g_ MO, which is the d_z2_ AO of nickel. Despite the similar arrangement of the MOs according to the results of PBE and B97D calculations, in the first case, the ruffling-distorted structure with χ_ruf_ = 17.8° corresponds to the minimum, and in the second case, the structure of the molecule is flat. Schemes of some of the top occupied MOs are shown in Figure 6.

**Figure 7:**
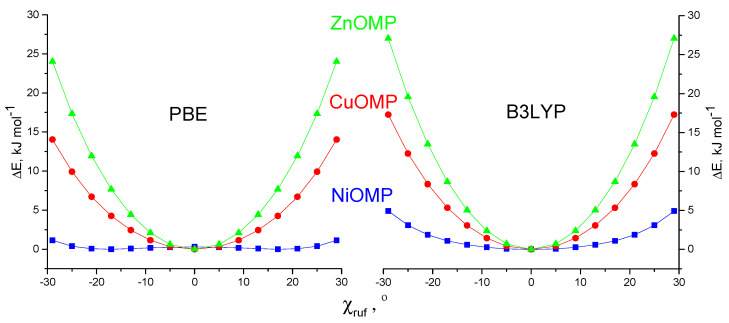
PES scans along χ_ruf_ = χ(C_α_-N∙∙∙N-C_α_) angle for NiOMP (squares), CuOMP (circles), ZnOMP (triangles) according to PBE/*cc-pVTZ* and B3LYP/*cc-pVTZ*.

In order to evaluate the nonrigidity of the NiOMP along the ruffling vibrational mode molecule in comparison with similar complexes of copper and zinc, the PES profiles of ruffling distortion U(C_1α_-N_1_-N_3_-C_6α_) were evaluated (Figure 7) by scanning the torsion angle χ(C_1α_-N_1_-N_3_-C_6α_) while optimizing all other geometric parameters at the PBE/cc-pVTZ and B3LYP/*cc-pVTZ* theory levels. The potential along the ruffling–vibrational coordinate in the case of NiOMP is rather soft, which creates a fundamental difficulty in the determination of the optimal geometry. According to the DFT calculations, NiOMP can be considered to be a quasi-planar molecule.

It should be noted that this uncertainty in the structure is also a feature of the parent NiP compound: the B3LYP/(*pVTZ*, *cc-pVTZ*) calculations predict a flat D_4h_ structure; in contrast, PBE calculations give the D_2d_ structure, but the D_4h_ structure (saddle point) has an energy that is only slightly higher than the energy of the D_2d_ structure (less than 1 kJ∙mol^−1^) (Table S2). According to the RI MP2 calculations, the NiP is characterized by a ruffling distortion with χ(C_α_-N··N-C_α_) = 33.7°, and the planar structure is higher in energy by 4.6 kJ∙mol^−1^. To gain a deeper insight into the problem, we made a single-point DLPNO-CCSD(T) calculation on the PBE/*pVTZ* (H, C, N), *cc-pVTZ* (Ni) geometries of NiOMP. According to the DLPNO-CCSD(T) calculations, the D_2d_ structure of NiOMP turned out to be more stable than the D_4h_ one by 0.6 kJ∙mol^−1^.

### 3.2. The Nature of Ruffling Distortion

**Figure 8:**
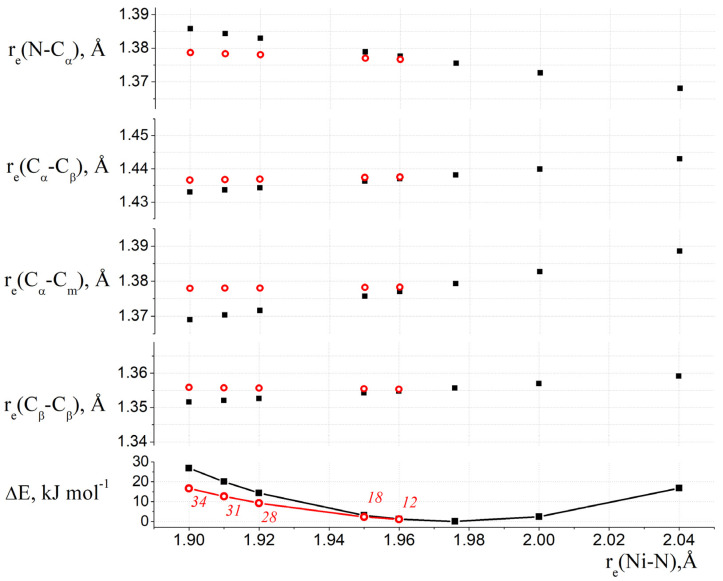
Internuclear distances N-C_α_, C_α_-C_m_, C_α_-C_β_, C_β_-C_β_ and relative energies of the structures vs. the internuclear distance of Ni-N in the NiP molecule from the B3LYP calculations with the *pVTZ* (H, C, N) and *cc-pVTZ* (Ni) basis sets. Red circles—for ruffling distorted structures, black squares—for flat structures. Red italics numbers indicate values of χ_ruf_ = χ(C_α_-N∙∙∙N-C_α_) for ruffling-distorted structures.

To simplify a description of the nature of the ruffling distortion in the porphyrin macrocycles, we considered the simplest nickel porphyrin, NiP. The QC calculations (B3LYP and PBE functionals, and basis sets of *pVTZ*—for H, C, N, *cc-pVTZ*—for Ni) of the NiP molecule were carried out for both for the planar (D_4h_) structures and for ruffling-distorted (D_2d_) structures at fixed values of the Ni-N bond lengths ranging from 1.90 Å to 2.04 Å, with an optimization of all other geometric parameters. Based on the obtained results, the dependence of the structural parameters of the macrocycle on the length of the Ni-N bond was depicted in Figure 8.

It is obvious that complexation is accompanied by a rearrangement of the porphyrin core. Thus, the equilibrium state is achieved as a compromise between the change in the macrocyclic cavity, which is necessary for optimal bond strength forming, and the forced macrocycle’s rearrangement. In the case of the B3LYP calculations, for r_e_(Ni-N) ≤ 1.96 Å, the studied planar structures correspond to saddle points on the PES. With an increase in the internuclear distance of Ni-N in the planar complex, the lengths of the C_α_-C_β_, C_α_-C_m_ and C_β_-C_β_ bonds increase along with the shortening of the N-C_α_ bond. An increase in the Ni-N distance to 1.976 Å leads to a decrease in the energy of the molecule; i.e., it is accompanied by the stabilization of the entire complex. With a further increase in the Ni-N distance, an increase in the amount of energy occurs. The most energetically favorable structure is characterized by the following values of the bond lengths: r_e_(C_α_-C_β_) = 1.438 Å, r_e_(C_β_-C_β_) = 1.356 Å, r_e_(C_α_-C_m_) = 1.379 Å, r_e_(N-C_α_) = 1.375 Å.

Figure 6 shows that the ruffling distortion makes it possible to almost achieve energetically favorable values of internuclear distances in a macrocycle with a shorter bond length between the Ni and N atoms. In this case, the energetically favorable values of internuclear distances N-C_α_, C_α_-C_m_, C_α_-C_β_, C_β_-C_β_ are achieved due to an increase in the ruffling angle C_α_-N∙∙∙N-C_α_ and correspond to the Ni-N distance being in the range of 1.90–1.96 Å. It should be recalled that these B3LYP calculations predict a flat structure (D_4h_) of the macrocycle. Similar dependences were obtained from the analysis of the results of the PBE calculations (Figure S1) with the only differences being that: (a) the ruffling-distorted structure of NiP (D_2d_) with an internuclear distance r_e_(Ni-N) = 1.956 Å corresponds to the minimum on the PES and (b) a planar structure with an internuclear distance r_e_(Ni-N) = 1.969Å has the lowest energy among the considered flat models and lies above the ruffling-distorted structure.

### 3.3. The Determination of NiOMP Structure Using the GED Method

The non-rigidity of the molecule along the ruffling coordinate is expressed by the shape of the PES along the dihedral angle χ(C_1α_-N_1_-N_3_-C_6α_) and leads to a low frequency of the corresponding vibration (Table 3 and Table 4). The same was found for a saddle-shaped distortion in normal mode. Therefore, according to the B3LYP/*pVTZ* (H, C, N), *cc-pVTZ* (Ni) calculations, ω_ruf_ = 8 cm^−1^, ω_sad_ = 20 cm^−1^. Within the framework of the SHRINK program formalism, such low frequencies lead to overestimated values of some of the vibrational corrections used in structural analysis. Thus, the vibrational corrections for the Ni–N distance and the distances between the nonbonded atoms of the same pyrrole ring, calculated on the basis of the results of the B3LYP calculations, are 0.03–0.07 Å. This leads to a strong disagreement between the theoretical and experimental molecular scattering functions sM(s) within the least squares (LS) procedure (Table 3, variants no. 5 and no. 6). In these cases, the structure of the porphyrin cycle is strongly distorted, and the difference between the r_h1_ and r_e_ parameters for the internuclear distances between the bound atoms reaches an unrealistic value of 0.02–0.10 Å.

Since the disagreement factor R_f_ obtained in the LS analysis using quantum chemical frequencies did not decrease below ~16%, we searched for the values of the wave numbers ω_ruf_, ω_sad_ corresponding to the minimum value of R_f_. For this purpose, an LS analysis was carried out repeatedly using the vibrational characteristics calculated using the vibration frequencies ω_ruf_, ω_sad_, which varied within the range of 8–100 cm^−1^. Table 3 summarizes the main results obtained using two variation schemes (fixing the ruffling angle obtained in the quantum chemical calculations and varying it) for different vibration frequencies ω_ruf_, ω_sad_.

According to the LS analysis performed with the use of scheme II (Table 3) (ω_ruf_ = 28 cm^−1^, ω_sad_ = 19 cm^−1^, PBE/*pVTZ* (H, C, N) *cc-pVTZ* (Ni)), the distance between the nickel and nitrogen atoms is r_h1_ (Ni-N) = 1.952 Å, the angle χ (C_α_-N∙∙∙N-C_α_) characterizing the degree of distortion is 14.6° and the disagreement factor is R_f_ = 4.32%. Freezing the quantum chemical value χ(C_α_-N∙∙∙N-C_α_) = 21.7° increases R_f_ to 4.98%, with the distances between the bonded atoms of the porphyrin core change not exceeding 0.002 Å. The optimal values of the vibration frequencies ω_ruf_ = 70 cm^−1^, ω_sad_ = 19 cm^−1^, found during their optimization, can reduce the disagreement down to R_f_ = 4.24%.

At the final stage of the structural refinement of NiOMP, the results of six variants of the LS analysis (1–4, 7, 8), shown in Table 3, were compared. The distances between the bonded atoms obtained during these variations are in good agreement with each other. Some difference in the value of the internuclear distance obtained with different variants of the LS analysis is observed for C_β_-C_β_. This is due to the difference between the quantum chemical differences [r(C_α_-C_β_)–r(C_β_-C_β_)] obtained in the PBE and B3LYP calculations and fixed during the LS analysis. The minimum disagreement factor is achieved in variant 4 (Table 3).

**Figure 9:**
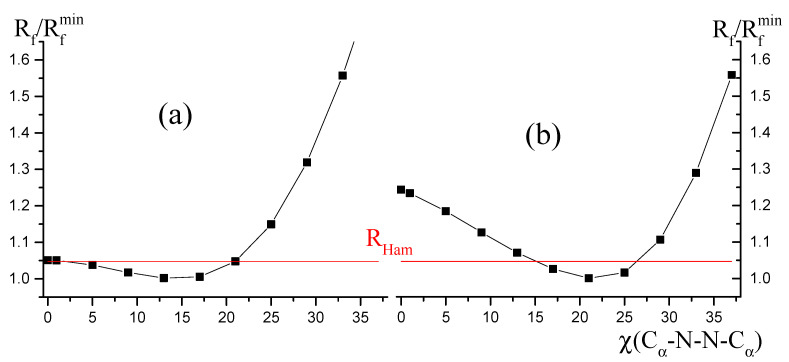
The ratio of disagreement factors R_f_/R_f min_ as a function of the torsion angle C_α_-N∙∙∙N-C_α_, responsible for ruffling distortion: (**a**)–using ω_ruf_ = 28 cm^−1^ and ω_sad_ = 19 cm^−1^, (**b**)—ω_ruf_ = 70 cm^−1^ and ω_sad_ = 19 cm^−1^. R_f_–factor of disagreement between experimental and theoretical molecular scattering intensities sM(s); R_f min_–minimal value of disagreement factor obtained in the LS analysis; R_Ham_–uncertainty according to Hamilton’s statistical criterion [53] at significance level 0.05.

Based on the Hamilton statistical approach [53], it was checked whether the flat and ruffling-distorted structures of the NiOMP molecule are distinguishable. For this purpose, additional LS calculations were performed according to variants 2 and 4 (Table 3), in each of which the value of the dihedral angle χ(X_1_-Ni-N_1_-C_1α_) was fixed. Figure 7 shows the relationship between the ratio R_f_/R_f,min_ of the disagreement factors and the ruffling angle χ (C_6α_-N_3_-N_1_-C_1α_), where R_f min_ is disagreement factor obtained in the LS analyses 2 and 4 (Table 3). In the first case (Figure 9a), the disagreement between experiment and theory decreases with a change in χ(C_6α_-N_3_-N_1_-C_1α_) from 0° to 15° and rapidly increases with a further increase in this parameter. Since the LS analysis was carried out using 377 experimental points (s = 1.2 − 15.3 Å^−1^, 2.8 − 26.2 Å^−1^ with a step of Δs = 0.1 Å^−1^) and 22 independent parameters, the value of the Hamilton statistical criterion at a significance level of 0.05 is R_Ham_ = R_f_/R_f, min_ = 1.047. In the case of a planar molecule, that is, at χ(C_6α_-N_3_-N_1_-C_1α_) = 0°, the ratio R_f_/R_f, min_ = 1.051 is almost equal to the Hamilton criterion R_Ham_ (Figure 9a). In the case of using variant 4 of the LS analysis (Table 3), the ratio R_f_/R_f, min_ = 1.24 at χ(C_6α_-N_3_-N_1_-C_1α_) = 0° significantly exceeds the Hamilton criterion (Figure 7), which formally indicates a substantial preference of the ruffling-distorted structure for the NiOMP molecule over a flat one.

Additionally, an alternative approach to the GED inverse problem solution with the GedModule program [54] was used. This one involves the variation of the force constants scale factors used for the amplitudes and vibration corrections calculations instead of the variation of the vibrational amplitudes in the classic version of GED LS analysis. The details of the approach are described in ref. [54]. In the LS structural analysis, the geometric parameters and the force field obtained in the calculations PBE/*pVTZ* (structure of D_2d_ symmetry) and B3LYP/*pVTZ* (planar structure of D_4h_ symmetry) were taken as starting values. It should be noted that the use of the force field obtained for the planar structure in the B3LYP/*pVTZ* calculations leads to slightly distorted structures (Table S3), whereas the structural analysis using a force field from PBE/*pVTZ* calculations gives a more distorted structure (Table S3).

Proving either the presence or absence of ruffling distortion using the GED method is a nontrivial task. Although, the fundamental difference in the shape of the dependences of R_f_/R_fmin_ on χ(C_α_-N∙∙∙N-C_α_) for the CoEP-II molecule [47] and for the NiOMP molecule makes it possible to state that the structure of NiOMP determined in the GED experiment is ruffling distorted.

In Table 4, the calculated structural parameters of NiOMP are compared with the experimental parameters of different nickel porphyrins obtained via XRD [28,29,30,31,34,52] and GED. The Ni-N internuclear distance of the free NiOMP molecule turned out to be almost equal to similar parameters according to an XRD analysis for ruffling-undistorted complexes. Despite this fact, it is necessary to bear in mind that the structural parameters obtained via the GED and XRD methods have different physical meanings [45]. The Ni–N bond length in the distorted NiOEP complex [34] is shorter by 0.02–0.03 Å than in the planar nickel porphyrins in the crystalline phase and NiOMP in the gas phase. The shortening of the Ni-N distances favors the strengthening of the bonding between the Ni and N atoms, and in turn explains the observed ruffling distortion.

## 4. Materials and Methods

### 4.1. Synthesis

For this process, 50 mg (0.118 mmol) of 2,3,7,8,12,13,17,18-octamethylporphine was washed into a boiling solution of 0.5 g (1.94 mmol) of acetylacetonate nickel (II) in 30 mL of tetrachloroethane. After the complete washout, the solution was boiled for another 1 h and cooled. Water and 5 mL of acetic acid were added to the mixture and the solvent was distilled off with steam, the precipitate was filtered, washed with distilled water and dried in air at 70 °C. The yield was 20 mg (35.4%). UV-vis λ_max_, nm (lg ε): 553 (4.46); 517 (4.93); 391 (5.28) (tetrachloroethane).

### 4.2. Combined Gas-Pase Electron Diffraction/Mass-Spectrometric Experiment

The combined gas-phase electron diffraction and mass spectrometric experiment was performed using the GED/MS apparatus [55,56]. An accurate wavelength of the electrons was determined from the diffraction patterns of polycrystalline ZnO. A sample of NiOMP was evaporated at 666(10) K, as measured by a W–Re 5/20 thermocouple, from a molybdenum effusion cell. The diffraction patterns were recorded on the Kodak Electron Image films SO-163 of 9 × 12 cm^2^ size at two nozzle-to-plate distances (598 and 338 mm). The conditions of GED/MS experiments are listed in Table 5. The optical densities of exposed films were recorded on a computer-controlled MD-100 (Carl Zeiss, Jena, GDR) setup [57]. For each film, a rectangular area of 10 × 130 mm^2^ was scanned in a diagonal direction (33 equidistant lines with a step of 0.1 mm along each line).

Simultaneously with registrations of the electron diffraction patterns, the mass spectra of the NiOMP vapors were recorded. The mass spectrum (Table S1) is typical for porphyrin complexes and is characterized by two groups of peaks corresponded to singly and doubly charged ions. Each group consisted of the parent ion and ions formed by consecutive removal of -CH_3_ groups. The recorded mass spectra contain no ions with a mass exceeding the mass of a single-charged molecular ion, which indicates the absence of dimeric forms and heavy volatile impurities. The mass spectra recorded during two independent experiments (with nozzle-to-plate distances of 338 and 598 mm) demonstrate a good reproducibility of the ion current relative abundances.

### 4.3. Quantum Chemical Calculations

Within the current investigation, QC calculations of the molecular geometries and Hessian of the NiOMP were performed using the Gaussian 09 program package [58] within the framework of the DFT method with the following functionals: B3LYP, PBE, PBE0, M06 and B97D. In this work, a large number of calculations were carried out using various basis sets: (a) 6–31G * [59,60,61,62]; (b) *pVTZ* [61,63,64,65] for C, N, H, *cc-pVTZ* [51] for Ni; (c) *cc-pVTZ* [51,64] for all atoms; (d) *cc-pVTZ*–for C, N, H, for describing the Ni atom, the effective core potential ECP10MDF [49] was used in combination with the basis (8s7p6d2f1g)/[6s5p3d2f1g] [48,49], taken from the site of the Stuttgart–Cologne group [66]. The QC calculations of the optimization geometric structure of the nickel porphyrins were performed using the program Priroda 9 [67] within the framework of the RI MP2 method and with the basis set L2.

The sophisticated DLPNO-CCSD(T) method [68,69,70], as implemented in the ORCA 4.0 code [71] for single-point calculations, was applied. The “TightPNO” DLPNO settings (TCutPairs = 10^−5^, TCutPNO = 10^−7^ and TCutMKN = 10^−3^) were used, as recommended, for applications where the most accurate values are targeted [72]. The sub-valence electrons on the nickel atom were correlated following the new ORCA 4 defaults [73,74]. The scalar relativistic effects were accounted for in the second-order scalar relativistic Douglas–Kroll–Hess (DKH2) Hamiltonian [75]. The following all-electron, relativistically recontracted [76], triple-ζ correlation-consistent basis sets were utilized in the present work. Dunning’s cc-pVnZ-DK basis sets were applied to describe the hydrogen, carbon and nitrogen atoms [50]. The nickel atom was described with the correlation-consistent, polarized, weighted core-valence cc-pwcVTZ-DK basis sets of Balabanov and Peterson [51]. The correlation fitting basis sets def2-qzvpp/C developed by Hättig [77,78], required for the resolution of the identity (RI) approximation as a part of DLPNO scheme, were used.

### 4.4. Structural Analysis

The analysis of the electron diffraction intensities was performed using a modified KCED-35 program, which is similar to the program described in the paper [79]. The model of NiOMP (Figure 2) was described through 26 independent parameters (X_1_ is a dummy atom defining the z axis direction and X_1_ is perpendicular to the molecule):

Ten internuclear distances: Ni-N_1_, N_1_-C_1α_, C_1α_-C_1β_, C_1β_-C_2β_, C_2α_-C_1m_, C_1m_-H_1m_, C^Me1^-C_1β_, H_1_^Me1^-C^Me1^, H_2_^Me1^-C^Me1^, H_3_^Me1^-C^Me1^;

Seven bond angles: Ni-N_1_-C_1α_, N_1_-C_1α_-C_1β_, H_4m_-C_4m_-C_1α_, C^Me1^-C_1β_-C_1α_, H_1_^Me1^-C^Me1^-C_1β_, H_2_^Me1^-C^Me1^-C_1β_, H_3_^Me1^-C^Me1^-C_1β_;

Nine torsion angles: X_1_-Ni-N_1_-C_1α_, C_1β_-C_1α_-N_1_-Ni, C_1β_-C_1α_-C_2β_-N_1_, C_4m_-C_8α_-C_1α_-Ni, H_4m_-C_4m_-C_8α_-C_1α_, C^Me1^-C_1β_- C_1α_-N_1_, H_1_^Me1^-C^Me1^-C_1β_-C_1α_, H_2_^Me1^-C^Me1^-C_1β_-H_1_^Me1^, H_3_^Me1^-C^Me1^-C_1β_-H_1_^Me1^.

The angles N_1_-Ni-X_1_, N_4_-N_i_-X_1_-N_1_ are fixed at 90° and the dihedral angles N_3_-Ni-X_1_-N_1_ are fixed at 180°.

In least-squares (LS) analysis, two schemes for the refinement of the structural parameters were carried out (see Table 6). In the first scheme, the coordinates determining the ruffling distortion of the macrocycle were not refined. The second scheme implies the refinement of coordinates describing the ruffling distortion. These independent geometric parameters were refined simultaneously with 13 groups of vibrational amplitudes corresponding to the different peaks on the radial distribution curve. The starting values of the vibration amplitudes and the vibrational corrections to the internuclear distances were calculated using the SHRINK program [80,81,82], as well as the starting values of the geometric parameters were taken from the results of the B3LYP and PBE calculations using the following basis sets: for H, C, N–Dunning basis sets [63,64] supplemented by polarized functions *pVTZ* [61,65] (the set is referred to as the «GAMESS *pVTZ*» in the EMSL basis set exchange [83,84]), for Ni-*cc-pVTZ* [51]. Our earlier studies [44,46] revealed that QC calculations using this combination of basis sets give good agreement with the experimental results at a comparatively low cost of computational resources.

## 5. Conclusions

Ruffling distortion can manifest itself in porphyrin molecules when the central atom reduces the coordination cavity. This type of distortion reduces the changes in the internuclear distances in the porphyrin core that occur during complexation. The Ni-N distance is short enough and, therefore, the manifestation of ruffling distortion is possible in porphyrin complexes with Ni(II).

Theoretical calculations do not provide an unambiguous answer concerning the presence of ruffling distortion in the NiOMP molecule. The complexity of these studies is associated with the non-rigidity of the molecule along the “ruffling” coordinate.

Taking into account the shallow potential function of NiOMP along the ruffling coordinate, proving the presence or absence of ruffling distortion using the GED method is a challenging problem. Within this work, a large number of approaches to structural analysis were used. They differ from each other according to (a) the starting geometric parameters obtained by the different QC calculations, (b) the vibrational corrections and the starting vibrational amplitudes, (c) the scheme of independent variations of the molecular parameters and (d) the variants of the force field variations during structural analysis. Nevertheless, a comprehensive comparison of the GED results obtained for NiOMP, CuOMP [43] and CoEP-II [47], specifically the fundamental difference in the shape of the dependences of R_f_/R_fmin_ on distortion coordinate, allows us to state that the ruffling distortion takes place in the NiOMP free molecule.

Eventually, the ruffling distortion should be expected for the porphyrin metal complexes with M-N distance shorter than 1.96 Å.

## Figures and Tables

**Table 1:** Geometry parameters of NiOMP according to B3LYP calculations.

Basis Set	6–31G *^,^ ^a^		*pVTZ* ^b^	*cc-pVTZ* ^c^	*cc-pVTZ* ECP10MDF ^d^
Equilibrium Structure	D_4h_	D_2d_	D_4h_	D_4h_	D_4h_
ω_ruf_^e^, cm^−1^	21(i)	30	8	15	12
ΔE ^f^, kJ∙mol^−1^	1.38	0.00			
χ(C_α_-N∙∙∙N-C_α_),°	0.0	24.1	0.0	0.0	0.0
r_e_(Ni-N), Å	1.958	1.942	1.977	1.974	1.972
r_e_(N N), Å	2.769	2.746	2.796	2.792	2.789
r_e_(N-C_α_), Å	1.379	1.378	1.374	1.371	1.371
r_e_(C_α_-C_β_), Å	1.447	1.448	1.448	1.446	1.445
r_e_(C_α_-C_m_), Å	1.379	1.381	1.379	1.377	1.376
r_e_(C_β_-C_β_), Å	1.365	1.367	1.363	1.361	1.361
φ_e_(C_α_-C_m_-C_α_),°	123.9	123.4	124.6	124.6	124.5

^a^ 6–31G *—for all atoms*;*^b^ H, C, N—*pVTZ*, Ni—*cc-pVTZ*; ^c^ H, C, N, Ni—*cc-pVTZ*; ^d^ H, C, N—*cc-pVT*Z, Ni–ECP10MDF and (8s7p6d2f1g)/[6s5p3d2f1g][48,49]; ^e^ frequency related with ruffling distortion of macroheterocycle; ^f^ relative energy.

**Table 2:** Structural parameters of NiOMP according to DFT calculations, exploiting different functionals ^a^.

DFT-Functionals	B3LYP	PBE0	M06	B97D	PBE	
	D_4h_	D_4h_	D_4h_	D_4h_	D_4h_	D_2d_
ω_ruf_ ^b^, cm^−1^	15	7	13	11	14(i)	21
ΔE, kJ∙mol^−1 c^					0.30	0.00
χ(C_α_-N···N-C_α_),°	0.0	0.0	0.0	0.0	0.0	17.8
r_e_(Ni-N), Å	1.974	1.959	1.956	1.976	1.967	1.957
r_e_(N···N), Å	2.792	2.770	2.767	2.794	2.781	2.768
r_e_(N-C_α_), Å	1.371	1.366	1.366	1.379	1.381	1.380
r_e_(C_α_-C_β_), Å	1.446	1.440	1.437	1.446	1.448	1.448
r_e_(C_α_-C_m_), Å	1.377	1.373	1.371	1.380	1.380	1.381
r_e_(C_β_-C_β_), Å	1.361	1.358	1.355	1.367	1.370	1.370
φ_e_(C_α_-C_m_-C_α_),°	124.6	124.0	124.0	124.3	124.0	123.7

^a^*cc-pVTZ* (H, C, N, Ni) basis set [50,51]; ^b^ frequency related to ruffling distortion of macroheterocycle; ^c^ relative energy.

**Table 3:** The degree of ruffling distortion according to the different schemes of structural refinement.

Variants of LS Analysis	VS ^a^	Starting Parameters ^b^	ω_ruf_, cm^−1^	ω_sad_, cm^−1^	R_f_, %	χ_ruf_, deg.	r_h1_ (Ni-N), Å
1	I	D_2d_–PBE	28 ^c^	19 ^c^	4.98	21.7	1.953(4)
2	II	D_2d_–PBE	28 ^c^	19 ^c^	4.32	14.6(46)	1.952(4)
3	I	D_2d_–PBE	70 ^d^	19 ^d^	4.27	21.7	1.948(4)
4	II	D_2d_–PBE	70 ^d^	19 ^d^	4.24	22.0(36)	1.948(4)
5	I	D_4h_–B3LYP	8 ^c^	20 ^c^	16.93	0.0	2.056(11)
6	II	D_4h_–B3LYP	8 ^c^	20 ^c^	16.63	7.0(290)	2.073(12)
7	I	D_4h_–B3LYP	28 ^d^	20 ^d^	4.45	0.0	1.954(4)
8	II	D_4h_–B3LYP	28 ^d^	20 ^d^	4.40	10.4(64)	1.954(4)

^a^ Scheme of independent variation of molecular parameters: I—χ_ruf_ was not refined, and was fixed at the calculated value, II—χ_ruf_ was refined; ^b^ the method for calculation of vibrational amplitudes and corrections; ^c^ calculated values; ^d^ refined values.

**Table 4:** Structural parameters of nickel porphyrins according to QC, GED and XRD.

Parameters	NiP	NiOMP.					NiEP-I	NiTMP	NiOEP		
	XRD ^a^	RI MP2 ^b^	B3LYP ^c^	PBE ^c^	PBE ^c^	GED ^d^, r_h1_	XRD ^e^	XRD ^f^	XRD ^e^	XRD ^e^	XRD ^e^
	[31]	D_2d_	D_4h_	D_4h_	D_2d_	R_f_ = 4.24%	[52]	[30]	[34]	[28]	[29]
χ(C_α_-N···N-C_α_),°	1.6	32.6	0.0	0.0	21.7	22.0(36)	0.0	0.9	31.8	0.8	1.3
r(Ni-N), Å	1.951	1.897	1.977	1.970	1.956	1.948(4)	1.957	1.953	1.930	1.952	1.958
r(N···N), Å	2.759	2.683	2.796	2.786	2.766	2.755(6)	2.770	2.763	2.729	2.760	2.769
r(N-C_α_), Å	1.379	1.375	1.374	1.383	1.382	1.380(4)	1.396	1.384	1.386	1.385	1.376
r(C_α_-C_β_), Å	1.435	1.438	1.448	1.450	1.451	1.451(3)	1.427	1.439	1.448	1.444	1.444
r(C_α_-C_m_), Å	1.371	1.377	1.379	1.382	1.384	1.384(3)	1.406	1.378	1.372	1.364	1.371
r(C_β_-C_β_), Å	1.347	1.365	1.363	1.372	1.373	1.373(3)	1.335	1.334	1.363	1.332	1.346
r(C_β_-C^Me^), Å	-	1.490	1.497	1.496	1.496	1.508(4)	1.553	-	-	-	-
r(C_β_-C^Et^), Å	-	-	-	-	-	-	1.553	-	1.501	1.504	1.495
r(C_1_-C_2_), Å	-	-	-	-	-	-		-	1.507	1.526	1.506
φ(Ni-N-C_α_),°	127.8	128.1	127.6	127.8	127.7	127.8(2)	127.6	127.6	127.5	128.0	128.0
φ(C_α_-N-C_α_),°	104.3	103.9	104.9	104.3	104.7	104.4(4)	104.8	104.8	105.1	104.1	103.9
φ(C_α_-C_m_-C_α_),°	123.5	122.3	124.5	124.0	123.6	123.3(5)	118.4	121.9	124.1	125.2	125.2
φ(C_α_-C_β_-C_β_),°	106.8	106.1	106.3	106.3	106.3	106.1(4)	107.9	107.4	106.8	107.2	106.5

^a^ Average values, T = 127 K; ^b^ basis set L2; ^c^ basis set *pVTZ* (H, C, N), *cc-pVTZ* (Ni); ^d^ uncertainties for the bond lengths were estimated as [(2.5σ_LS_)^2^ + (0.002r)^2^]^1/2^ and for the bond angles as 3σ_LS_; ^e^ average values, T = 295 K; ^f^ average values, T = 140 K.

**Table 5:** Parameters ^a^ of the GED/MS experiment in synchronous mode for NiOMP.

L, mm	598	338
I, μA	0.96	1.37
U_acc_, kV	71	73
T, K;	663(10)	668(10)
p, Torr	2.9·10^−6^	2.0·10^−6^
t, s	45	98
N	6	6
s_min_-s_max_^b^, Å^−1^	1.2–15.3	2.8–26.2

^a^ L–nozzle-to-film distance, I–primary electron beam current, U_acc_–accelerating voltage (approximate), T–effusion cell temperature, p–residual pressure in diffraction chamber, t–exposure time, N–number of recorded films; ^b^ step Δs = 0.1 Å^−1^.

**Table 6:** Two schemes of the independent variation of molecular parameters in the LS refinement of NiOMP.

Molecular Parameters	Schemes of Independent Variation of Molecular Parameters	
	I	II
Bond lengths	Ni-N, N-C, C-C ^a^	Ni-N, N-C, C-C ^a^
Bond angles	Ni-N_1_-C_1α_, N-C_1α_-C_1β_	Ni-N_1_-C_1α_, N_1_-C_1α_-C_1β_
Torsion angles	-	X_1_-Ni-N_1_-C_1α_, C_4m_-C_8α_-C_1α_-Ni

^a^ the differences between C_α_-C_β_, C_β_-C_β_, C_α_-C_m_ bond lengths were fixed at the calculated values.

## Data Availability

Not applicable.

## Supplementary Materials

The following supporting information can be downloaded at https://www.mdpi.com/article/10.3390/ijms23010320/s1.
