# Supplementary material for: Molecular Structure of Nickel Octamethylporphyrin—Rare Experimental Evidence of a Ruffling Effect in Gas Phase
Source: Int J Mol Sci. 2021 Dec 28;23(1):320. doi: 10.3390/ijms23010320 (PMC8745403; doi:10.3390/ijms23010320)
Supplement: Supplementary file 1 [file ijms-23-00320-s001.zip › FINAL after proofreading/NiOMP-Supplement.pdf]

Supplementary Materials for

Molecular Structure of Nickel Octamethylporphyrin—Rare Experimental Evidence of a Ruffling Effect in Gas Phase

Alexander E. Pogonin [1,*], Arseniy A. Otlyotov [2,3], Yury Minenkov [3,4], Alexander S. Semeikin [5], Yuriy A. Zhabanov [2], Sergey A. Shlykov [6] and Georgiy V. Girichev [2,*]

1 Department of Nanomaterials and Ceramic Technology, Ivanovo State University of Chemistry and Technology, Sheremetevsky Avenue 7, 153000 Ivanovo, Russia; pogonin@isuct.ru (A.E.P.)
2 Department of Physics, Ivanovo State University of Chemistry and Technology, Sheremetevsky Avenue 7, 153000 Ivanovo, Russia; arseney_otlyotov@mail.ru (A.A.O.); zhabanov@gmail.com (Y.A.Z.), girichev@isuct.ru (G.V.G.)
3 N.N. Semenov Institute of Chemical Physics of Russian Academy of Sciences, Kosygina Street 4, 119991 Moscow, Russia; Yury.Minenkov@chph.ras.ru
4 Joint Institute for High Temperatures, Russian Academy of Sciences, 13-2 Izhorskaya Street, Moscow 125412, Russia
5 Department of Organic Chemistry, Ivanovo State University of Chemistry and Technology, Sheremetevsky Avenue 7, 153000 Ivanovo, Russia; semeikin@isuct.ru (A.S.S.)
6 Department of Physical and Colloidal Chemistry, Ivanovo State University of Chemistry and Technology, Sheremetevsky Avenue 7, 153000 Ivanovo, Russia; shlykov@isuct.ru (S.A.S.)
* Correspondence: pogonin@isuct.ru (A.E.P.); girichev@isuct.ru (G.V.G.); Tel.: +7-4932-30-0960 (A.E.P.); Tel.: +7-4932-35-9874 (G.V.G.)

Table S1. Mass-spectra of NiOMP saturated vapor.

Mass/charge	Ion	Intensity, %
478	$[C_{28}H_{28}N_4Ni]^+$	100
463	$[C_{27}H_{25}N_4Ni]^+$	23.4
448	$[C_{26}H_{22}N_4Ni]^+$	7.8
433	$[C_{25}H_{19}N_4Ni]^+$	2.7
239	$[C_{28}H_{28}N_4Ni]^{2+}$	14.6
232	$[C_{27}H_{25}N_4Ni]^{2+}$	12.7
224	$[C_{26}H_{22}N_4Ni]^{2+}$	15.5
217	$[C_{25}H_{19}N_4Ni]^{2+}$	10.2
209	$[C_{24}H_{16}N_4Ni]^{2+}$	4.8
202	$[C_{23}H_{13}N_4Ni]^{2+}$	1.5

Table S2. Structural parameters of NiP and NiOMP according to QC calculations.

	NiP								NiOMP						
	B3LYP pVTZ[a]	B3LYP cc-pVTZ[b]	PBE pVTZ[a]		PBE cc-pVTZ[b]		RI MP2[c]		B3LYP pVTZ[a]	B3LYP cc-pVTZ[b]	PBE pVTZ[a]		PBE cc-pVTZ[b]		RI MP2[c]
	D_{4h}	D_{4h}	D_{4h}	D_{2d}	D_{4h}	D_{2d}	D_{4h}	D_{2d}	D_{4h}	D_{4h}	D_{4h}	D_{2d}	D_{4h}	D_{2d}	D_{2d}
ω_{ruf} [d], cm^{-1}	19.7	26.1	25.5(i)	37.0	18.9(i)	28.9			7.8	15.3	19.6(i)	27.9	14.4(i)	20.7	
ΔE [e], $kJ\cdot mol^{-1}$			0.7	0.0	0.2	0.0	4.6	0.0			0.9	0.0	0.3	0.0	
$\chi(C_\alpha\text{-N}\cdot\cdot\text{N-}C_\alpha)$,°	0.0	0.0	0.0	20.9	0.0	17.3	0.0	33.7	0.0	0.0	0.0	21.7	0.0	17.8	32.6
r_e(Ni-N), Å	1.976	1.973	1.969	1.956	1.966	1.957	1.932	1.898	1.977	1.974	1.970	1.956	1.967	1.957	1.897
r_e(N···N), Å	2.795	2.790	2.785	2.767	2.780	2.768	2.732	2.684	2.796	2.792	2.786	2.766	2.781	2.768	2.683
$r_e(N\text{-}C_\alpha)$, Å	1.375	1.373	1.385	1.384	1.383	1.382	1.378	1.375	1.374	1.371	1.383	1.382	1.381	1.380	1.375
$r_e(C_\alpha\text{-}C_\beta)$, Å	1.438	1.436	1.440	1.441	1.438	1.439	1.430	1.431	1.448	1.446	1.450	1.451	1.448	1.448	1.438
$r_e(C_\alpha\text{-}C_m)$, Å	1.379	1.377	1.383	1.385	1.381	1.382	1.373	1.378	1.379	1.377	1.382	1.384	1.380	1.381	1.377
$r_e(C_\beta\text{-}C_\beta)$, Å	1.356	1.353	1.363	1.364	1.361	1.362	1.356	1.358	1.363	1.361	1.372	1.373	1.370	1.370	1.365
$\varphi_e(C_\alpha\text{-N-}C_\alpha)$,°	105.0	105.0	104.4	104.8	104.4	104.7	103.3	104.2	104.9	104.9	104.3	104.7	104.3	104.6	103.9
$\varphi_e(N\text{-}C_\alpha\text{-}C_\beta)$,°	110.8	110.8	111.1	110.9	111.1	111.0	112.0	111.4	111.2	111.2	111.6	111.3	111.6	111.4	111.9
$\varphi_e(C_\alpha\text{-}C_m\text{-}C_\alpha)$,°	124.0	124.0	123.6	123.2	123.5	123.3	123.0	122.0	124.5	124.6	124.0	123.6	124.0	123.7	122.3
$\varphi_e(C_\alpha\text{-}C_\beta\text{-}C_\beta)$,°	106.7	106.7	106.7	106.7	106.7	106.7	106.3	106.5	106.3	106.3	106.3	106.3	106.3	106.3	106.1

[a] H, C, N – *pVTZ*, Ni – *cc-pVTZ*; [b] H, C, N, Ni – *cc-pVTZ*; [c] L2 basis set; [d] frequency related with ruffling distortion of macroheterocycle; [e] relative energy.

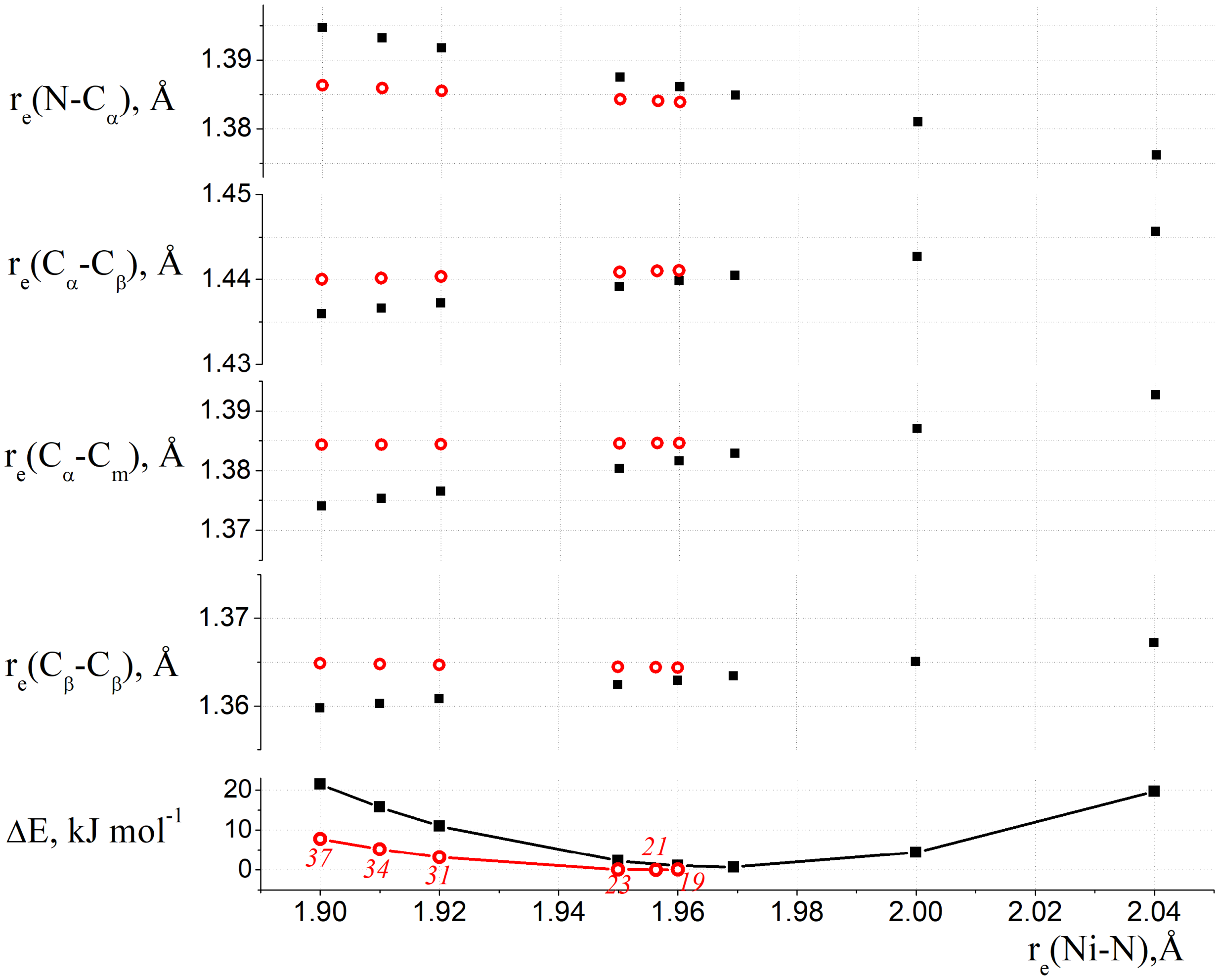

Figure S1. Dependence of internuclear distances N-C_α, C_α-C_m, C_α-C_β, C_β-C_β and the relative energies of structures on the internuclear distance Ni-N in the NiP molecule according to PBE calculations using basis sets pVTZ (H, C, N) and cc-pVTZ (Ni). Red circles - for ruffling distorted structures, black squares - for flat structures. Red italics numbers indicate values of $\chi_{ruf}=\chi(C_\alpha\text{-N}\cdots\text{N-}C_\alpha)$ for ruffling distorted structures.

Table S3. Molecular parameters (r_{h1}) of NiOMP, obtained with different starting geometries and force fields using GedModule program.

	1	2	3	4
Starting geometric parameters	PBE [a]	B3LYP [b]	B3LYP [b]	PBE [a]
Starting Force Field	PBE [a]	PBE [a]	B3LYP [b]	B3LYP [b]
R_f, %	4.82	4.44	5.50	5.55
r(Ni-N), Å	1.956(4)	1.960(6)	1.968(52)	1.969(48)
$\chi(C_\alpha\text{-N}\cdots\text{N-}C_\alpha)$,°	18.1(3.9)	4.5(8.9)	3.2(3.1)	1.7(13.3)
r(N-C_α), Å	1.381(4)	1.384(8)	1.378(18)	1.380(19)
r(N···N), Å	2.766(6)	2.772(9)	2.783(73)	2.784(68)
r(C_α-C_β), Å	1.448(4)	1.448(4)	1.453(21)	1.450(27)
r(C_β-C_β), Å	1.382(4)	1.379(4)	1.384(20)	1.384(25)
r(C_β-C_β), Å	1.371(4)	1.364(4)	1.369(20)	1.373(25)
r(C_β-C^{Me}), Å	1.510(7)	1.507(5)	1.496(93)	1.497(106)
φ(Ni-N-C_α),°	127.8(1)	128.0(4)	127.8(8)	127.9(7)
φ(C_α-N-C_α),°	111.6(7)	111.7(6)	112.0(1.1)	112.0(1.3)
φ(C_α-C_m-C_α),°	123.9(2)	123.9(9)	123.7(2.7)	123.9(2.7)
ω_{ruf} [c], cm^{-1}	23	17	16	16
ω_{sad} [d], cm^{-1}	31	26	27	26

[a] ruffling distorted structure (D_{2d} symmetry) from PBE calculation with *pVTZ* (H, C, N), *cc-pVTZ* (Ni); [b] planar structure (D_{4h} symmetry) from B3LYP calculation with *pVTZ* (H, C, N), *cc-pVTZ* (Ni); [c] recalculated value of frequency related with ruffling distortion of macroheterocycle at the end of structural analysis; [d] recalculated value of frequency related with saddling distortion of macroheterocycle at the end of structural analysis.
